# The Rapid Degradation of Lambda-Cyhalothrin Makes Treated Vegetables Relatively Safe for Consumption

**DOI:** 10.3390/ijerph15071536

**Published:** 2018-07-20

**Authors:** Rousseau Djouaka, Murielle Farrelle Soglo, Michael Olugbenga Kusimo, Razack Adéoti, Armand Talom, Francis Zeukeng, Armand Paraïso, Victor Afari-Sefa, May-Guri Saethre, Victor Manyong, Manuele Tamò, Jeff Waage, Jo Lines, George Mahuku

**Affiliations:** 1 International Institute of Tropical Agriculture (IITA), Cotonou 08 BP 0932, Benin; r.djouaka@cgiar.org (R.D.); r.adeoti@cgiar.org (R.A.); talomarmand@yahoo.fr (A.T.); zeusfranck07@yahoo.com (F.Z.); m.tamo@cgiar.org (M.T.); 2 Department of Nutrition and Food Sciences, Faculty of Agronomic Sciences, University of Abomey-Calavi, P.O. Box 526, Cotonou 08 BP 0932, Benin; 3 Center for Research in Infectious Diseases (CRID), Yaounde, Cameroon; gkusimo@gmail.com; 4 Department of Animal Biology, Faculty of Sciences, University of Dschang, P.O. Box 96, Dschang, Cameroon; 5 Department of Biochemistry, Faculty of Sciences, University of Yaounde I, P.O. Box 812, Yaounde, Cameroon; 6 Department of Vegetal Production, Faculty of Agronomy, University of Parakou, P.O. Box 123, Parakou, Benin; arparaiso@yahoo.fr; 7 World Vegetable Center, C/O IITA-Benin Campus, Cotonou 08 BP 0932, Benin; victor.afari-sefa@worldveg.org; 8 International Institute of Tropical Agriculture (IITA), PMB 5320, Ibadan 200284, Nigeria; m.saethre@cgiar.org; 9 International Institute of Tropical Agriculture (IITA), P.O. Box 34441, Dar-es-Salaam, Tanzania; v.manyong@cgiar.org (V.M.); G.Mahuku@cgiar.org (G.M.); 10 Department of Disease Control, Faculty of Infectious and Tropical Diseases, London School of Hygiene and Tropical Medicine (LSHTM), London WC1E 7HT, UK; jeff.waage@lshtm.ac.uk (J.W.); Jo.Lines@lshtm.ac.uk (J.L.)

**Keywords:** Benin, HPLC, lambda-cyhalothrin residues, vegetables

## Abstract

Lambda-cyhalothrin (λ-cyhalothrin) is the most commonly used pyrethroid insecticide for vegetable farming in Benin. This insecticide is misused and overused by farmers, and hence may pose health hazards to consumers. We monitored λ-cyhalothrin residues in lettuce and cabbage from farms at the market gates in Cotonou and Parakou using high performance liquid chromatography (HPLC) analysis techniques. These residues were also monitored on samples directly from farms (on-farm sampling) for 14 days post-treatment. Potential factors such as photolysis and hydrolysis involved in λ-cyhalothrin degradation were also screened. Results revealed that the level of λ-cyhalothrin residue concentrations in lettuce from Houeyiho decreased from 4.2 mg/kg on Day 1 to about 0.2 mg/kg on Day 7. On Day 9, analyzed lettuces were all λ-cyhalothrin free. In contrast, even 14 days after treatment of cabbage from Bawera (Parakou), we still recorded the presence of λ-cyhalothrin residues in analyzed samples. For samples from market gates, λ-cyhalothrin residues were found in lettuce from two markets out of the nine surveyed in Cotonou. Interestingly, none of these contaminated samples had residues above the maximum residue limit for lettuce (MRL = 0.5 mg/kg). Similarly, in Parakou, samples from all five surveyed vegetable markets were contaminated with λ-cyhalothrin residues at concentrations below the MRL for cabbage (MRL = 0.2 mg/kg). We conclude that λ-cyhalothrin residues in lettuce and cabbage from farms and markets in Parakou and Cotonou are within the MRL, and hence are relatively safe for consumption.

## 1. Introduction

Use of market gardening has been expanding in the Republic of Benin since its introduction as part of the national poverty alleviation strategy launched in the early 2000s [1,2]. Rapid population growth, especially in urban and peri-urban areas, has resulted in high food demands and has contributed to sustenance of this urban and peri-urban livelihood strategy.

As vegetable production is rapidly expanding, so is the prevalence of related inherent pests and vegetable diseases, and hence, there is a need for the application of more agro-chemical pesticides [2,3]. The introduction of synthetic pesticides in crop protection in general has significantly contributed to increased productivity [4]. Several classes of synthetic insecticides, such as organochlorides, organophosphates, carbamates, pyrethroids, neonicotinoids, and ryanoids are used in crop production. Of these various classes, pyrethroids remain the least toxic [5]. Pyrethroids are highly effective and several times more toxic to insects than mammals. This is because mammals have poor dermal absorption and rapidly metabolize them to non-toxic metabolites [6]. Although less toxic, the misuse and overuse of pyrethroids in crop production may lead to food contamination and this may constitute sources of health hazards for farmers and consumers [6,7]. Ingestion of pyrethroid residues may lead to sore throat, nausea, vomiting, abdominal pain, mouth ulcers, increased secretions, and/or dysphagia [6]. Extreme exposure can lead to coma and convulsions, conditions, which are life-threatening [7]. The misuse and overuse of pyrethroids could lead also to the resistance of infectious disease vectors (such as *Anopheles gambiae*, which is involved in malaria) due to the repetitive exposure of those vectors to the insecticide residue in farming areas [8]. 

The maximum residue limit (MRL) of pesticides for each commodity is determined by the Codex Alimentarius Commission (CAC). The MRL serves as one of the quality control parameter for protecting the health of consumers of agricultural products while facilitating international trade [9]. MRL standards are used worldwide to promote safer food and maintain Acceptable Daily Intake of pesticides.

Previous reports on vegetable farming in Benin have highlighted the indiscriminate use of pesticides by farmers [10,11,12]. λ-cyhalothrin is one approved pyrethroid insecticide for pest control in vegetable farming in Benin [13]. This insecticide is said to be the most commonly used by vegetable farmers. However, few studies have attempted to screen residues of this insecticide on vegetables. To analyze the relative safety of this insecticide for vegetable protection, we monitored the degradation of λ-cyhalothrin in lettuce (*Lactuca sativa*) and cabbage (*Brassica oleracea*) from farms at the market gates in Cotonou and Parakou, two major cities in Benin.

## 2. Materials and Methods

### 2.1. Study Sites

**Figure 1:**
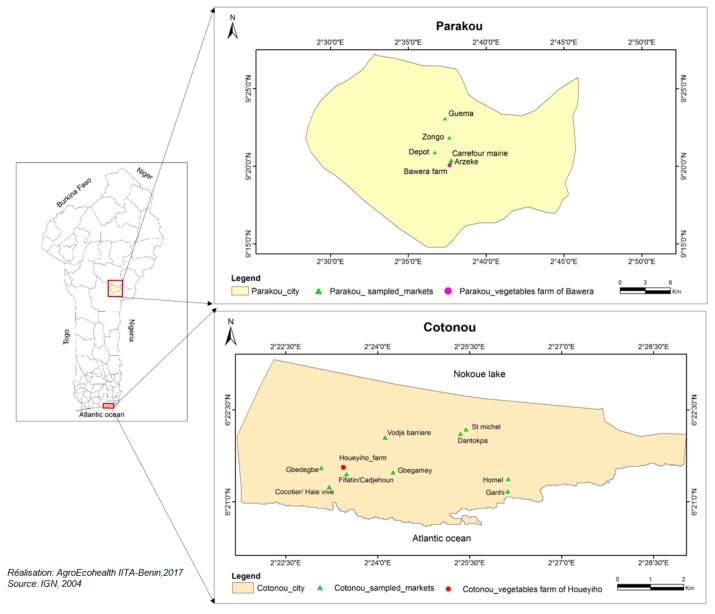
Map of sampling sites in the cities of Cotonou and Parakou in the Republic of Benin.

This study was conducted in two cities of Benin, namely Cotonou (6°36′ N–2°41′ E, Altitude: 20 m) in the south and Parakou (9°31′ N–2°63′ E, Altitude: 350 m) in the north. Two main target vegetable farms and surrounding sales collection points, located in Houeyiho and Bawera, were surveyed. The Houeyiho and Bawera farms were selected as they are the largest vegetable farms of the cities (Cotonou and Parakou, respectively). Moreover, the Bawera farm is close to the largest market in Parakou; the Arzeke market [14,15]. Vegetable markets also surveyed include: Gbedegbe, Fifatin, Gbegamey, St. Michel, Cocotier, Dantokpa, Homel, Vodje barrier, and Ganhi in Cotonou, and Arzeke, Zongo, Depot, Guema, and Carrefour Mairie in Parakou (Figure 1).

### 2.2. Pesticide Utilization in Vegetable Farming

Surveys were carried out from March to May 2016 at the Houeyiho and Bawera vegetable farms and sales collection points. Relevant information on the use of pesticides by farmers was gathered through focus group discussions and direct field observations and complemented with one-on-one interviews to producers via the aid of structured questionnaires administered. Data collected included: the socio-demographic characteristics of farmers (e.g., age, sex and social status of farmers), type of pesticides used by vegetable farmers, pesticide dosage levels, application rates, and frequencies of usage. Based on the number of farmers in both sites, we determined the minimal acceptable size of farmers to be interviewed. In both studied sites, at least 10 percent of farmers were interviewed as published by Agueh et al. [16]. Hence, 40 voluntary farmers in Bawera and 41 in Houeyiho were involved in the survey after obtaining their consents.

### 2.3. Handling and Transportation of Vegetables from Farms to Markets

Four (4) focus group discussions (FGDs), each with an average size of 10 participants, were conducted in November 2016 with vegetable sellers to investigate the origin of water used for the pre-cleaning of vegetables, the containers used for vegetables packaging, the level of exposure of produce to sunlight, the means of transport to the market, and the means of storage of vegetables from the farm through to the market and until sale of produce to the final consumers. In addition to the structured interviews, we conducted direct observations in the market on vegetable sellers to better describe the extent of hygienic handling practices for lettuce and cabbage in Cotonou and Parakou.

### 2.4. Collection of Lettuce and Cabbage from Target Farms (Houeyiho and Bawera) for λ-Cyhalothrin Residue Analysis

Lettuce and cabbage collections were performed between October and November 2016, dates which correspond to the rainy season in Benin. The average temperatures during this period were 26.4 °C and 27 °C, respectively, for October and November. It was observed and mentioned by farmers that repeated insecticide applications are required in the rainy season as most applications are washed away by rains. Lettuces and cabbages were considered in Cotonou and Parakou, respectively, because of the relatively high demands of both vegetables in the two cities. Most farmers indicated λ-cyhalothrin as their insecticide of choice in vegetable protection. Hence, insecticide-treated lettuces and cabbages were collected and analyzed one day (D1) after treatments. The treatment was done with “lambda super 2.5 EC” which contains 25 g/L of λ-cyhalothrin. Other samples were collected from the same farm at intervals of 3 days till the 14th day after insecticide treatment for monitoring λ-cyhalothrin residues on leaves. The 14-day timeframe used in this monitoring corresponds to the time allowed by farmers for vegetable harvest after the last pesticide treatment. For each marked day, composite samples of lettuce and cabbage were collected from Houeyiho and Bawera, respectively, leading to a total of six composite samples from each vegetable production site. No rainfall was recorded during the sampling and screening period. Collected samples were well-labeled, wrapped in aluminum foil, stored in coolers, and transported to the laboratory where they were kept at 4 °C for analysis. Prior to the analysis, composite samples were divided into three replicates. We confirmed the use of λ-cyhalothrin through direct observation of empty containers of this insecticide in surveyed farms. Although we cannot entirely exclude the possibility that some farmers might use other insecticides apart from λ-cyhalothrin, we focused this first part of our research on the monitoring of λ-cyhalothrin residues as the main insecticide used by vegetable farmers.

### 2.5. Collection of Lettuce and Cabbage from Market Gates in Surveyed Towns (Cotonou and Parakou) for λ-Cyhalothrin Residue Analysis

Lettuce and cabbage on sale at the surveyed market gates (Figure 1) in Cotonou and Parakou were collected for λ-cyhalothrin residue analysis before purchase by consumers for household consumption. In each market (nine in Cotonou and five in Parakou), samples of vegetables were collected from three vegetable sellers; about 500 g of vegetable was collected from each seller. The three samples were pooled together to constitute one composite sample per market. Collected vegetable samples were wrapped in aluminum foil, stored in coolers, and transported to the laboratory where they were kept at 4 °C for analysis.

### 2.6. Collection of Water Samples from Water Bodies Found within Vegetable Farms for λ-Cyhalothrin Residue Analysis

Water bodies found around the vegetable farm of Houeyiho in Cotonou were aseptically collected in glass bottles. Five water samples from the five identified standing water bodies were bottled. These bottles were stored in coolers and brought to the laboratory where they were kept at 4 °C for pesticide residue analysis.

### 2.7. Processing of Samples in the Laboratory for Detection of λ-Cyhalothrin Residues Using HPLC

#### 2.7.1. Extraction of Insecticide Residues from Collected Samples (Lettuce, Cabbage, Water Mixed with Soil Particles)

For each sample of vegetable to be analyzed, about 100 g was chopped and mixed. From this mixture, 1 g was weighed and ground with 1 mL HPLC technical grade acetonitrile (Sigma Aldrich, St. Louis, MI, USA). The slurry was vigorously shaken on a vortex for 20 min and centrifuged for 20 min at 10,000 rpm for phase separation. The supernatant was collected and re-centrifuged for 15 min at 10,000 rpm; an aliquot of 75 μL was then loaded into HPLC vials for quantification of λ-cyhalothrin residues.

With regard to the collected water, 1.5 L of each sample was filtered, and sieved particles were dried and well homogenized. The dried particles were analyzed using modified protocol of El-Saeid et al. [17]. One gram of the dried particles was weighed and mixed with 1 mL HPLC technical grade acetonitrile (Sigma Aldrich, St. Louis, MI, USA). The mixture was gently microwaved five times at 30 s each per session using a SEKOM–SM-720CPZ microwave (Sekom, Shenzhen, China) at the medium power rate. The slurry generated from this set of intermittent microwaving was vigorously shaken on a vortex for 20 min and centrifuged for 20 min at 10,000 rpm for phase separation. The supernatant was collected and re-centrifuged for 15 min at 10,000 rpm; an aliquot of 75 μL was then loaded into HPLC vials for quantification of λ-cyhalothrin residues in collected water samples mixed with soil particles.

#### 2.7.2. Quantification of λ-Cyhalothrin Residues in Collected Samples after Extraction

λ-cyhalothrin was quantified from each 75-μL aliquot of samples as previously described by Togola et al. [18] using a reverse phase HPLC machine Agilent technology 1260 infinity (Agilent Technologies Deutschland GmbH & Co. KG, Waldbronn, Germany). The HPLC column used was C18, 5 µm 120 Â, 4.6 × 250 mm (Thermo Fisher scientific, Waltham, MA, USA), and the mobile phase (HPLC grade solutions, Sigma Aldrich) was a mixture of methanol and water (90:10). The flow rate of 1 mL/min was maintained and the sample injection volume was 50 µL. The elution was monitored with HPLC UV detector at 226 nm. The chromatographic peaks corresponding to the retention times in the column for each sample were identified and compared with the determined retention time of λ-cyhalothrin standard solution. The λ-cyhalothrin concentration in each sample was later calculated using an equation generated from the standard curve.

#### 2.7.3. Method Validation: λ-Cyhalothrin Standard Curve Plotting, Computation of Limit of Detection (LOD) and Limit of Quantification (LOQ) of the HPLC, and Recovery

**Figure 2:**
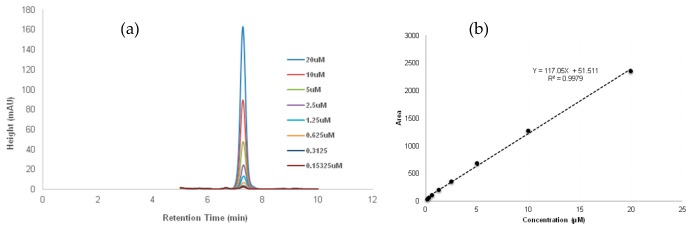
Standard curve of λ-cyhalothrin. (**a**) The chromatogram overlay of the serial dilutions of the insecticide; (**b**) The standard curve plotted between peak areas and the various concentrations of the insecticide.

Serial dilutions from a 20-µM stock solution of commercialized λ-cyhalothrin (Sigma Aldrich) were constituted, loaded into HPLC vials, and analyzed using the reverse-phase HPLC machine (Agilent technology 1260 infinity, Germany). The HPLC procedure was performed as above. The standard curve was then plotted between the insecticide concentrations (independent value) and the areas of the peaks (dependent value) generated from the chromatogram. The LOD and LOQ of the λ-cyhalothrin were determined using the linear regression method as previously described by Shrivastava and Gupa [19]. The quality of pesticides was assured through the analysis of solvent blanks, procedure blanks and duplicate samples. The method was optimized and validated using spiked samples together with the internal standard to evaluate the recovery of compounds. The retention time of λ-cyhalothrin for this analysis was 7.20 min and the regression equation generated was y = 117.05x + 51.511 with a correlation coefficient of 0.9979 (Figure 2). The LOD and LOQ were 0.009 mg/L and 0.028 mg/L, respectively. Percentages of recovery were 64% and 84%, respectively, for lettuce and cabbage.

### 2.8. Screening of Potential Factors of λ-Cyhalothrin Degradation

#### 2.8.1. Effect of Photolysis on λ-Cyhalothrin Degradation

To investigate the effect of photolysis on λ-cyhalothrin degradation, on-farm growing lettuces were sprayed with λ-cyhalothrin insecticides (lambda super 2.5EC). After spraying, some lettuce plants were immediately collected, and the concentration of insecticide residue quantified using HPLC techniques. Other samples taken from the same set were wrapped with aluminum foil and stored in the fridge (5 °C) where they were protected from rays of light. Both sets of samples (on-farm growing vegetables and fridge-stored vegetables) were monitored periodically for λ-cyhalothrin residues for a total of 15 days. Each sample was divided in three parts (triplicate) and separately analyzed. The rate of degradation of λ-cyhalothrin on growing lettuce was then compared with that of lettuce wrapped and protected from light.

#### 2.8.2. Effect of Ultra Violet (UV) Light (Direct Exposure) on λ-Cyhalothrin Degradation

To further establish the effect of UV (photolysis) on λ-cyhalothrin degradation, 15 samples containing lambda stock solution 20 µM (9 mg/L) were prepared in transparent glass vials and then exposed to direct UV light 8 W, 302 nm (UVP, Cambridge, UK) at various time intervals (20 min, 6 h, 12 h, and 24 h). Unexposed sample were kept as control. λ-cyhalothrin residues in each vial were quantified using HPLC techniques.

#### 2.8.3. Effect of Alkaline Solutions (Hydrolysis) on λ-Cyhalothrin Degradation

The effect of alkalinity on λ-cyhalothrin degradation in lettuce was also investigated. Lettuce was liquefied using HPLC-grade water and the potential hydrogen (pH) of the solution recorded. Liquefied lettuce, HPLC grade water, and NaOH solutions of pH 10 and pH 12 were all spiked with stock solution of λ-cyhalothrin (20 µM). This experiment was conducted in triplicate and samples were incubated overnight at room temperature. Lambda-cyhalothrin residue concentrations were determined for each sample (liquefied lettuce, HPLC grade water, and NaOH solutions of pH 10 and pH 12) using HPLC techniques.

## 3. Results

### 3.1. Pesticides Utilization by Farmers at Houeyiho and Bawera

**Figure 3:**
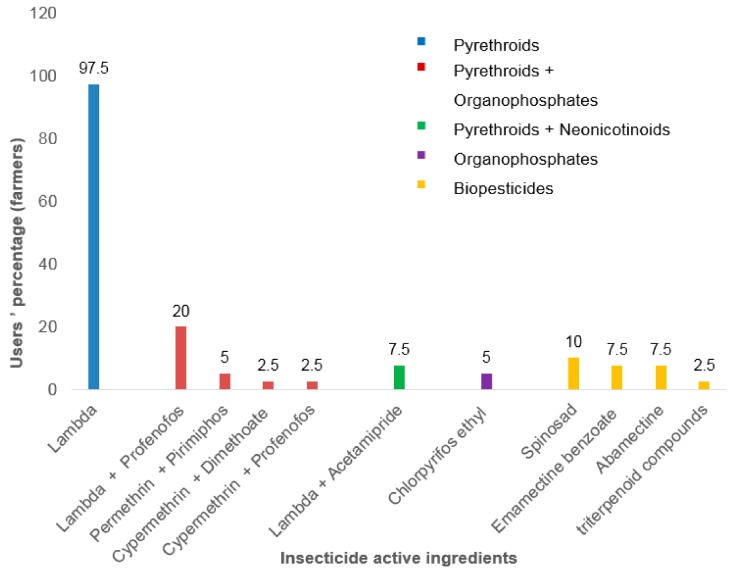
Insecticide families used for vegetable treatments in Houeyiho.

**Figure 4:**
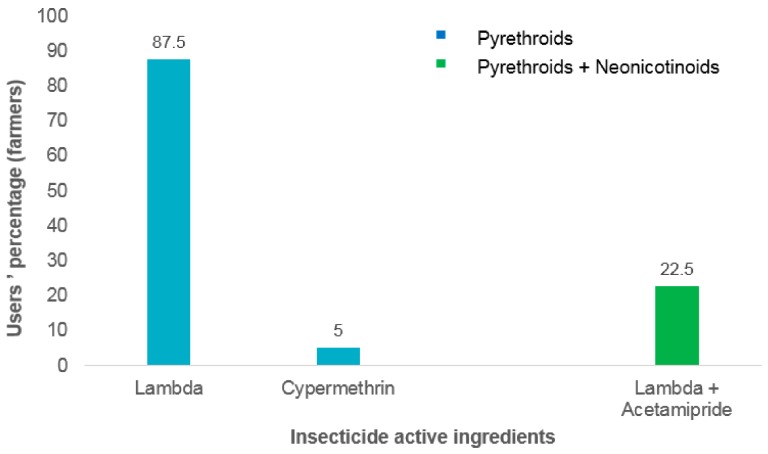
Insecticide families used for vegetables treatment in Bawera.

Surveys conducted in the two selected vegetable farms revealed that pyrethroids are the most frequently used insecticides in Houeyiho and Bawera (Figure 3 and Figure 4). Pyrethroid insecticides are either used alone or in combination with other insecticides families such as organophosphates or neonicotinoids. Among the pyrethroids used by farmers, λ-cyhalothrin is the main active ingredient used in Houeyiho farm, with a usage proportion of 95.1% (Figure 3). In Bawera, λ-cyhalothrin was used by 87.5% of farmers (Figure 4).

Vegetable treatments with λ-cyhalothrin vary in both surveyed farms. The majority of farmers in Bawera (91.4%) apply treatments with λ-cyhalothrin insecticide (lambda super 25EC) at intervals of 7 days, whereas the majority of farmers in Houeyiho (51.3%) observe intervals of 14 days in between treatments (Table 1).

It was also recorded that treatment concentrations with λ-cyhalothrin vary from one farmer to another and from one site to another site. In Bawera, recorded treatment concentrations were 5 mL of λ-cyhalothrin in 10 L of water (concentration observed by 34.3% of farmers), 2.5 mL/10 L (concentration observed by 51.4% of farmers) and 7.5 mL/10 L (for about 14.3% of farmers). In Houeyiho, most farmers (56.4%) followed the recommended dilution of 5 mL of the insecticide in 10 L, as indicated in the manufacturer label.

### 3.2. Vegetable Handling from Farms to Market Gates

Qualitative investigations (FGDs) conducted with farmers and vegetable sellers revealed that vegetables are sold on farms and sellers collect fresh vegetables directly from farmers. At times, farmers instead supply sellers with fresh vegetables at the market gates. Before vegetables are transported from the farms to the market, they are usually washed and rinsed with the irrigation water found within the vicinity of farming areas. After washing, vegetables are well-packed in plastic bags, jute bags, or baskets, or wrapped with cloth materials, and transported to market gates. In the two surveyed cities (Cotonou and Parakou), wrapped vegetables are typically transported to market gates by motorcycle or car.

### 3.3. On Farm Monitoring of λ-Cyhalothrin Residues in Growing Vegetables Post Treatment

**Figure 5:**
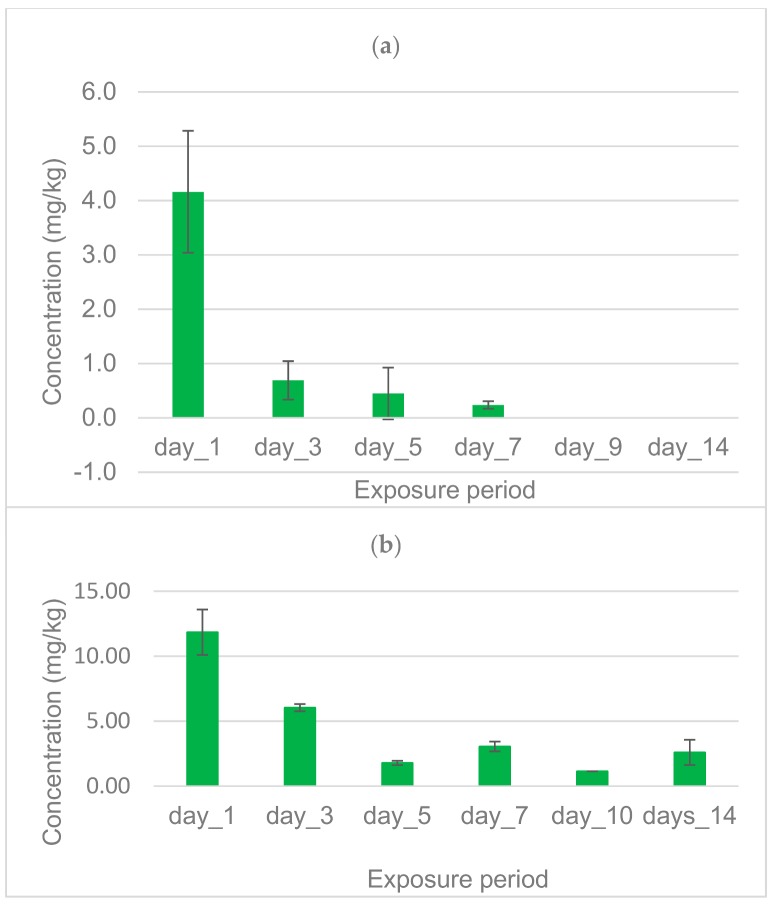
On-farm monitoring of λ-cyhalothrin residues on growing vegetables after insecticide application: (**a**) distribution of λ-cyhalothrin residues on lettuces from Houeyiho; (**b**) Distribution of λ-cyhalothrin residues on cabbages from Bawera.

In Houeyiho, the concentration of λ-cyhalothrin in growing lettuce considerably decreased from 4.16 mg/kg on Day 1 after insecticide application to 0.24 mg/kg on Day 7. From Day 9 to Day 14 after application of λ-cyhalothrin on lettuce, no trace (0 mg/kg) of this insecticide was found on harvested and analyzed leaves (Figure 5a). On the Bawera farm, 11.84 mg/kg of λ-cyhalothrin residue was detected in growing cabbages on Day 1 post treatment. This concentration decreased to 3.05 mg/kg on Day 7. On Day 10 and Day 14, we recorded the presence of λ-cyhalothrin residues on harvested cabbage at concentrations of 1.14 mg/kg and 2.60 mg/kg, respectively (Figure 5b).

### 3.4. Screening of Pesticide Residues in Water Bodies Found in Vegetable Farms at Houeyiho

Out of the five water bodies mapped in the vegetable farm of Houeyiho in Cotonou, residues of λ-cyhalothrin were detected in two. The concentrations of λ-cyhalothrin residues detected ranged from 0 (no detection of the λ-cyhalothrin) to 0.277 mg/kg (Table 2).

### 3.5. Screening of λ-Cyhalothrin Residues in Lettuces and Cabbages from Vegetable Markets of Cotonou and Parakou

Insecticide residues were monitored in lettuce and cabbage samples from the most active vegetable markets in Cotonou and Parakou. λ-cyhalothrin residues were detected in lettuce collected from two (22.22%) markets (Ganhi and Homel) out of the nine surveyed in Cotonou (Table 3). Detected concentrations of λ-cyhalothrin residues on lettuce samples from the two markets were below 0.002 mg/kg. In Parakou markets, cabbage samples collected from all the five (5) surveyed markets still carried λ-cyhalothrin residues. Detected concentrations of λ-cyhalothrin ranged from 0.12 mg/kg to 0.18 mg/kg. The highest concentration was recorded with samples from Arzeke market, while the lowest concentration was recorded with samples from the Depot market. Comparative analysis with the standard maximum levels (0.5 mg/kg for lettuce and 0.2 mg/kg for cabbages) revealed that levels of λ-cyhalothrin contamination of vegetables in Cotonou and Parakou remained below the MRL standards (Table 3).

### 3.6. Screening of Potential Factors of λ-Cyhalothrin Degradation

#### 3.6.1. Effect of Photolysis on λ-Cyhalothrin Degradation

**Figure 6:**
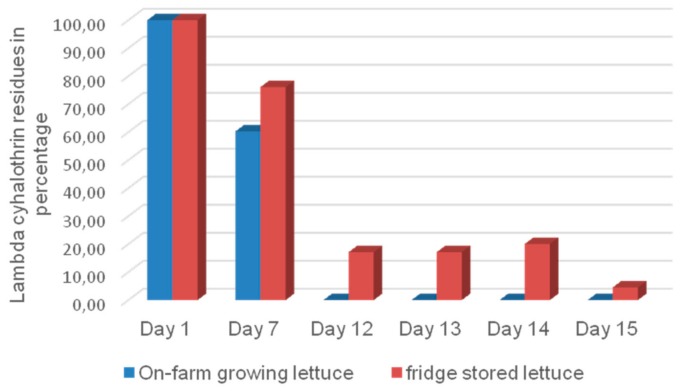
Distribution of λ-cyhalothrin residues on growing lettuces compared to fridge-stored lettuce.

λ-cyhalothrin in on-farm growing lettuce degraded completely after 7 days, whereas the rate of degradation was relatively low for treated lettuce stored in the fridge and protected from light (Figure 6).

#### 3.6.2. Effect of UV Light (Direct Exposure) on λ-Cyhalothrin Degradation

**Figure 7:**
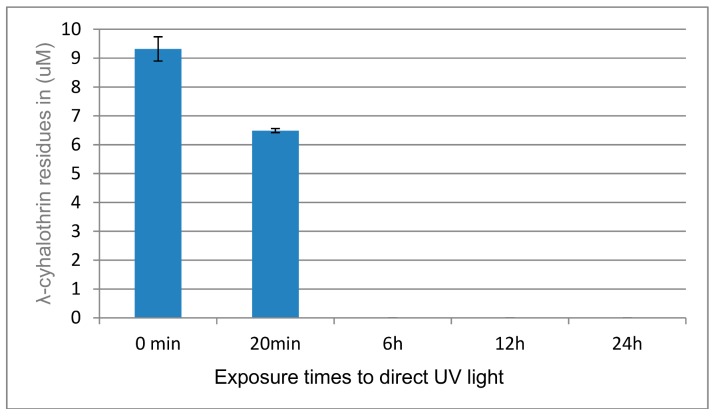
Quantification of λ-cyhalothrin residues at various time exposures to direct UV light.

**Figure 8:**
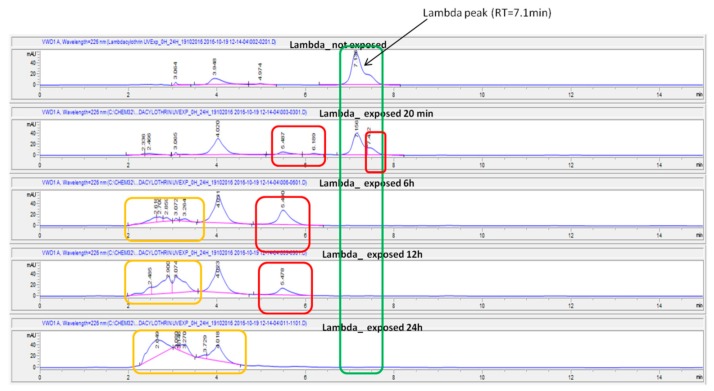
Chromatograms of the UV photolysis of λ-cyhalothrin at various exposure time intervals. The residues of λ-cyhalothrin are in the green box and the suspected metabolites in other boxes.

The degradation of λ-cyhalothrin exposed to direct UV (302 nm) was observed almost immediately, with 50% degradation recorded within 20 min (Figure 7). Two additional hydrophilic metabolites were noted at retention times (RTs) of 5.4 min and 6.1 min (Figure 8). No residue of λ-cyhalothrin was found after 6 h incubation under UV light. However, after 6-h, 12-h, and 24-h exposures, we rather observed a set of metabolites as shown in (Figure 8).

#### 3.6.3. Effect of Alkaline Solutions (Hydrolysis) on λ-Cyhalothrin Degradation

When λ-cyhalothrin was introduced into pH 10 and pH 12 solutions, no residue was found. In contrary, when same insecticide was added to HPLC grade water and liquefied lettuce, the recorded quantities of residues were respectively 2.1 µM and 2.2 µM (Table 4).

## 4. Discussion

Synthetic pesticides prevent losses and maintain good yield of agricultural products by protecting the crops from pest invasion [20]. However, when pesticides are indiscriminately used they can be toxic to humans and have negative effects on ecosystems, with beneficial insects inadvertently killed [21]. Some banned chemical pesticides are still widely used in developing countries where the implementation of regulations remains relatively poor [22]. We have investigated the use of chemical pesticides applied on vegetables and monitored in this study the residues of used chemical insecticides on lettuces and cabbages from farms at market gates.

### 4.1. Pesticides Utilization by Vegetable Farmers in Benin

This study highlights that vegetable farmers mostly use pyrethroid insecticides in Houeyiho and Bawera. Data generated from administered structured questionnaires revealed that 95.1% and 87.5% of market gardeners use λ-cyhalothrin for vegetable protection against pests in Houeyiho and Bawera vegetable farms, respectively. Similar results were highlighted by Agueh et al. [19] who reported that 82.4% of farmers in Houeyiho used λ-cyhalothrin for vegetable treatment. λ-cyhalothrin is an insecticide belonging to the pyrethroids group. This group of insecticides has been documented as less toxic to mammals, and hence can be encouraged for pest protection for commodities that can be eaten directly (raw consumption) like vegetables. Pyrethroid insecticides are among the list of authorized pesticides to be used in vegetable production in Benin because of their efficacy, their short lifespan, and their relatively low toxicity [13]. To reduce the documented misuse, abuse, or overuse of synthetic pesticides in vegetable faming [11,23], it was noted that some farmers in Houeyiho use botanical pesticides such as neem oil, especially during the latency period when vegetables have already reached maturity and are awaiting harvest.

### 4.2. Vegetable Handling from Farms to Market Gates

In this study, we observed that harvested vegetables are washed and rinsed in situ with irrigation before being taken to the market. The use of opened irrigation water bodies for washing and rinsing of vegetables is a practice which was also documented in the vegetable farms of Kumasi in Ghana [24]. In contrast, a washing system based on the use of clean tap water is highlighted in Accra, the capital town of Ghana [24]. Opened irrigation water bodies are usually contaminated with insecticides residues resulting from the application of pesticides on vegetables for pests’ control [25]. Akogbeto et al. [26] also reported that after pesticide treatments in agriculture sites, insecticide residues are washed by rainfall and sediment into watering pools located below cultivated areas. Similarly, we observed the presence of residues on water bodies sampled within the vegetable farm of Houeyiho in Cotonou. The use of irrigation water for washing and rinsing could increase the level of insecticide residues in vegetables. Even if vegetables were free from insecticide residues during harvest, they could eventually get contaminated with insecticide residues during washing/rinsing with irrigation water. Hence, the quality of water used by farmers for washing and rinsing of vegetables needs to be substantially improved through mass sensitization and awareness. The transportation of vegetables from the farm to the market is mostly done in this African region with motorbikes, tricycles, cars, or trucks [27]. Microbial contamination can occur if vegetables are transported in dirty cars or tricycles or are kept/wrapped in dirty bags. In all cases, vegetables should properly be washed with disinfectants at home prior to consumption.

### 4.3. On-Farm Analysis of λ-Cyhalothrin Residues in Growing Vegetables Post Last Treatment

In this study, we recorded a quick on-farm degradation of λ-cyhalothrin (which is the main insecticide used by farmers) during the latency period. For both lettuces and cabbages, we observed a natural degradation rate between Day 1 and Day 14 after the last-treatment (latency period) which was remarkable. On Day 7 for example, the level of λ-cyhalothrin detected on lettuce was lower than the MRL (0.5 mg/kg), making this vegetable product relatively safe for consumption on Day 7 after the last treatment. On the contrary, we recorded a persistence of λ-cyhalothrin on Day 14 in cabbage. Although the degradation rate was also remarkable with cabbages, the concentration of λ-cyhalothrin residues detected in this vegetable product on Day 14 was 2.60 mg/kg, a value far above the European commission MRL (MRL-Cabbage = 0.2 mg/kg) [28]. λ-cyhalothrin, like most pyrethroids, is known to degrade fast. This quick degradability makes it relatively safe for use in the environment [29]. Photolysis, hydrolysis, and microbes (*Pseudomonas* and *Serratia*) have been described as the main factors responsible for the degradation of pyrethroids insecticides [30,31,32,33,34,35]. In addition to mentioned external factors of degradation (sun rays and micro-organisms), plants are known to internally produce a higher amount of degradation enzymes, such as cytochrome P450 enzymes. The immobile nature of plants leads to their exposure to several xenobiotics, including target and non-target agro-chemicals [36]. The degradation enzymes thus serve as their means of defense against these toxic chemicals [29]. The recorded persistence of λ-cyhalothrin in cabbage compared to lettuce could be explained by their differential physical structures (lettuces are light in weight with few leaves while cabbages have a set of super-posed/compiled leaves) and also, the sensitivity of cabbage leaves to vegetable pests, which is attributed to its luxuriant and nutritive nature [37]. Therefore, this commodity is likely to receive more pesticide applications than lettuce, resulting in greater residues in cabbages compared to lettuce, as documented in this research. It is also possible that the folded pattern of cabbage could limit the effect of sunlight radiation, leading to slower rate of λ-cyhalothrin degradation in cabbages as compared to lettuces. A low degradation of λ-cyhalothrin in cabbage as compared to leafy vegetables (spinach) was reported also by Henry et al. [38] in Tanzania. Pest control in cabbage production should ideally require fewer agrochemical applications. The use of biological agents such as *Beauveria bassiana* should be encouraged [39]. The entomopathogenic fungus *B. bassiana* has shown to be effective against diamondback moth *Plutella xylostella* L. which is the most devastating pest for cabbage. This biological insecticide is environmentally friendly and harmless to humans [40,41].

### 4.4. Monitoring of λ-Cyhalothrin Residues In Lettuce and Cabbage on Sale at Vegetable Markets in Cotonou and Parakou

Following the analysis of lettuce sold at vegetable markets of Cotonou, we noticed a low presence of λ-cyhalothrin residues in samples from Ganhi and Homel. In both markets, the detected level of residue was <0.002 mg/kg, below the MRL standards for lettuce (0.5 mg/kg). This information on the very low contamination of lettuce by pesticide residues already obtained at the farm level is further confirmed at the market gates. In the process of handling/transportation from the farm to the market gates λ-cyhalothrin levels on lettuce significantly drop, and this insecticide is well degraded by the time lettuce arrives at the market, making this vegetable product relatively safe for consumption. Similarly, we noticed that relatively high concentrations of λ-cyhalothrin recorded in cabbages at farms levels significantly decreased when these vegetables arrived at the market. It was found that both lettuce and cabbages had λ-cyhalothrin residues below standard MRL based on market gates surveys. On the contrary, Sæthre et al. [2] showed that some specific families of vegetables such as *Solanum marcrocarpon* leaves (gboma) and tomatoes still accumulate pesticide residues at concentrations above standard MRL by the time they arrive at some market gates in Benin. In Ghana, Akomea-Frempong et al. [42] reported the low presence of λ-cyhalothrin residue in all sampled lettuce from farms, markets, street foods, cafeterias, and restaurants in Kumasi; the residue concentrations were all bellow the MRL.

Nevertheless, the repetitive consumption of vegetables with low levels of λ-cyhalothrin residue could in the long term be associated to health risk [6,7,42].

### 4.5. Screening of λ-Cyhalothrin Residues in Water Bodies Found in Vegetable Farms at Houeyiho

Λ-cyhalothrin residues were found in water pools identified within the vegetable farm of Houeyiho. It was noticed that these water pools were used for irrigation and also for the washing/rinsing of harvested vegetables. The presence of pesticide residues in this water source could signal a source for contamination of vegetables by pesticide residues. The presence of these water bodies could also favor the development of malaria vectors. It has been documented by Akogbeto et al. [26]; Djouaka et al. [23], and Yadouleton et al. [43] that *Anopheles* larvae populations which breed in these insecticide-contaminated water bodies might undergo high insecticide selection pressure and as a result develop resistance against public health insecticides commonly used for malaria vector control; the development of insecticide resistance by malaria vectors is a rapidly growing phenomenon which is an important public health concern.

### 4.6. Screening of Potential Factors of λ-Cyhalothrin Degradation

λ-cyhalothrin degradation was faster in on-farm growing lettuce than treated lettuce stored in the fridge and protected from light rays. The rapid degradation of this insecticide at farm level could be due to ultra violet (UV) rays from sunlight. This UV-linked degradation of insecticides could also be coupled with other mechanisms such as microbial, insect, and plant enzymatic decomposition through P450 enzymes as described by Niu [29]. The direct exposure of λ-cyhalothrin solution to UV light (302 nm) at different exposure times showed the complete degradation of this insecticide after 6 h; this further confirms the implication of UV light (photolysis) in the quick degradation of λ-cyhalothrin in on-farm growing vegetables as reported by. Fernandez-Alvarez et al. [44].

Hydrolysis is the other assessed mechanism of λ-cyhalothrin degradation [32]. It has been reported that pyrethroids are stable at pH below 8 and hydrolyze at pH above 9 via the nucleophilic reaction of the hydroxyl ion of alkalines [45]. In this study we showed that on-farm growing lettuce had a pH of 6, and therefore the hypothesis of pH-linked degradation of λ-cyhalothrin in lettuce could not be considered in this study.

## 5. Conclusions

This study on the quality of lettuces and cabbages in Benin showed that λ-cyhalothrin-treated vegetable products like lettuces and cabbages contain reasonable amounts of residues of this insecticide at farm levels. These residues significantly degrade and get to concentrations below the MRL when reaching market gates. Photolysis was identified in this research as a potential factor contributing to λ-cyhalothrin degradation. Although vegetables with insecticide residue levels below the MRL could be considered relatively safe for consumption, it is worth indicating that their bio-accumulation through regular consumption could constitute a health risk for consumers. It also appears that farmers can opt for a wide range of pesticides when facing population of pests resistant to standard insecticides, resulting in contamination by other types of insecticide residues in vegetables. Our study highlights the need to reduce chemical pesticide utilization in vegetable production for improved food safety. Further studies are being conducted to investigate the presence of other insecticide residues (aside from λ-cyhalothrin) on lettuces and cabbages sold in Benin markets.

## Figures and Tables

**Table 1:** Frequency and quantity of lambda super (λ-cyhalothrin) treatment in Houeyiho and Bawera.

Period of Insecticide Application		Houeyiho		Bawera	
		*n*	%	*n*	%
Frequency of insecticide treatment	Every 7 days	10	25.6	32	91.4
	Every 10 days	4	10.3	0	0.0
	Every 14 days	20	51.3	3	8.6
	Every 30 days	5	12.8	0	0.0
Insecticide concentration (dilution)	2.5 mL/10 L	15	38.5	18	51.4
	3 mL/10 L	2	5.1	0	0.0
	5 mL/10 L	22	56.4	12	34.3
	7.5 mL/10 L	0	0.0	5	14.3
Total		39	100	35	100

*n*: Number of respondents.

**Table 2:** Level of λ-cyhalothrin residues in water samples collected from mapped water bodies identified at the vegetable farm of Houeyiho.

Water Samples	λ-Cyhalothrin Residues (mg/kg)
WP 1	ND
WP 2	ND
WP 3	0.005 ± 0.008
WP 4	ND
WP 5	0.277 ± 0.090

ND: not detected; WP: water pool (identified standing water pools).

**Table 3:** λ-cyhalothrin levels in lettuces and cabbages sampled at vegetable markets in Cotonou and Parakou.

Sampled Cities	Market Name	Types of Vegetables	Mean (mg/kg) ± SD
Cotonou	Gbedegbe	Lettuce	0
	Gbegamey	Lettuce	0
	Ganhi	Lettuce	<0.002
	Homel	Lettuce	<0.002
	St Michel	Lettuce	0
	Dantokpa	Lettuce	0
	Cocotier/Haie vive	Lettuce	0
	Vodje barriere	Lettuce	0
	Fifatin/Cadjehoun	Lettuce	0
Parakou	Depot	Cabbage	0.120 ± 0.016
	Zongo	Cabbage	<0.002
	Arzeke	Cabbage	0.184 ± 0.020
	Guema	Cabbage	0.142 ± 0.037
	Mairie	Cabbage	0.113 ± 0.065

SD: standard deviation. Maximum residue limit (MRL) value for lettuce = 0.5 mg/kg; MRL value for cabbage = 0.2 mg/kg.

**Table 4:** Quantification of λ-cyhalothrin residues in alkaline solution.

	HPLC Grade Water	Liquefied Lettuce	NaOH Solution pH 10	NaOH Solution pH 12
**pH**	7	6	10	12
**λ concentration (μM)**	2.1	2.2	0	0
